# The classification of coronal deformity based on preoperative global coronal malalignment for adult spinal deformity is questionable

**DOI:** 10.1186/s12891-022-05246-4

**Published:** 2022-03-29

**Authors:** Jiandang Zhang, Yang Yu, Shangju Gao, Yong Hai, Bing Wu, Xiaojing Su, Zheng Wang

**Affiliations:** 1 Department of Orthopaedic Surgery, Beijing Chaoyang Hospital, Capital Medical University, Beijing, China; 2 Nankai University School of Medicine, Nankai District, 94 Weijin Road, Tianjin, 300071 China; 3 Department of Orthopaedic Surgery, Hebei General Hospital, 348 Hepingxi Road, Shijiazhuang, 050000 China; 4 Department of Spine Surgery, The 4th medical center of Chinese PLA General Hospital, 51 Fucheng Rd, Beijing, 100037 China

**Keywords:** Classification, Global coronal malalignment, Coronal imbalance, Degenerative lumbar scoliosis

## Abstract

**Study design:**

Retrospective case–control radiographic study.

**Objective:**

To identify main effects of preoperative pattern and global coronal malalignment (GCM) on postoperative coronal imbalance in degenerative lumbar scoliosis (DLS) patients and evaluate the rationality of the classification of coronal deformity based on preoperative GCM.

**Summary of background data:**

A classification of coronal deformity based on preoperative GCM (20 mm set as the threshold of coronal imbalance) has been proposed recently, but whether it is practical is unclear.

**Methods:**

One hundred twelve DLS patients treated with posterior instrumented fusion were reviewed. Coronal measurements included GCM and major Cobb angle. Based on relationship between C7 PL and major curve, preoperative patterns were classified into: Pattern 1(concave pattern), C7 PL shifted to the concave side of major curve; Pattern 2(convex pattern), C7 PL shifted to the convex side of major curve*. P*atients were separated into 4 groups (3 types): Type 0–1: GCM < 20 mm plus Pattern 1; Type 0–2: GCM < 20 mm plus Pattern 2; Type 1: GCM > 20 mm plus Pattern 1; Type 2: GCM > 20 mm plus Pattern 2. After comparison within patterns or among 4 groups, further factorial analysis was performed.

**Results:**

Significant differences regarding postoperative GCM or coronal imbalance/balance ratio existed among 4 groups (F = 6.219, *p* = 0.001; *x*^2^ = 22.506, *p* < 0.001, respectively), despite no significant difference in intra-pattern 1(concave pattern) or intra-pattern 2(convex pattern) groups. Two-way analysis of variance showed preoperative pattern exhibited significant effect on postoperative GCM or imbalance/balance ratio (F_(1,108)_ = 14.286, *p* < 0.001; F_(1,108)_ = 30.514, *p* < 0.001, respectively) while neither preoperative GCM alone nor interaction of preoperative GCM with pattern did.

**Conclusion:**

In DLS patients, it’s the preoperative pattern other than GCM that had main effects on postoperative coronal imbalance. Classification of coronal deformity based on preoperative GCM is questionable.

**Level of evidence:**

3

## Background

Degenerative lumbar scoliosis (DLS) is a common condition in older population with a reported prevalence ranging from 7.5% to 30% [1, 2]. Classifying adult scoliosis may facilitate decision-making based on the comparison of similar cases and provide guidance for treatment. Several different classifications have been described by Aebi [3], Lowe et al [4], Schwab et al [5] and provide spine surgeons with different information [6]. However, none of them focused on the impact of preoperative coronal deformity on the postoperative coronal imbalance.

Recently, Bao et al [7] proposed a novel classification of coronal deformity based on the absolute value of preoperative global coronal malalignment (GCM) and demonstrated that patients with type C [GCM greater than 30 mm and C7 plumb line (PL) shifted to the convex side of major curve) were at greater risk for postoperative coronal imbalance than type A (GCM less than 30 mm) and type B (GCM greater than 30 mm and C7 PL shifted to the concave side of major curve). More recently, Obeid et al [8] adhered to the same basic principle as Bao’s and proposed a similar but more extensive classification using 20 mm as the threshold of coronal imbalance. However, the big weakness of Obeid’s classification is that it was based on the authors’ personal experiences, not on clinical experiments. Therefore, it is unknown whether the classification is feasible or not. Moreover, it is still undetermined whether preoperative GCM or preoperative pattern based on spatial relationship between C7 PL and major curve had main effects on postoperative coronal imbalance. Since one of the aims of the classification is to help guide the treatment, once a classification is defective, following the classification might lead to disastrous complications.

The purpose of this study was to identify the main effects of preoperative pattern and GCM on the postoperative coronal imbalance in DLS patients and evaluate the rationality of classification of coronal deformity based on preoperative GCM by Obeid et al [8].

### Materials and methods

#### Patient population

This study was approved by Ethical Committee of our hospital, and all methods were performed in accordance with the guidelines and regulations of the ethics review board. We retrospectively collected consecutive DLS patients with age greater than 45 years between January 2015 and December 2019. Inclusion criteria included: primary spinal deformity correction and instrumented fusion through posterior-only approach. Exclusion criteria included: fusion levels < 5, history of hip or knee arthroplasty, absolute discrepancy of leg length > 20 mm [9]. 112 DLS patients were enrolled in this study eventually. Informed consent was obtained from all subjects.

#### Surgical techniques

After exposure, bilateral pedicle screws were inserted at every level in the construct. 84 patients were fused to pelvis while 28 patients were fused to L5. To obtain better deformity correction, spinal osteotomies including Schwab grade I (facetectomy) or grade II (Ponte osteotomy) were conducted in all patients [9]. Decompression and transforaminal lumbar interbody fusion (TLIF) were performed at the caudal part of lumbar spine (L2-S1) if assistive anterior support was necessary, or spinal stenosis was present [9].

#### Radiographic evaluation

Whole spine standing posteroanterior and lateral X-rays were collected before surgery and at discharge from hospital or 2 weeks after surgery. Surgimap (version 2.2.15; Spine Software, New York, NY) was used to perform the coronal and sagittal measurements by two independent spine researchers and the mean values were collected for analysis. Coronal measurements included: (1) GCM, defined as the horizontal distance between C7 PL and central sacral vertical line (CSVL) [7]; (2) major Cobb angle, defined as the angle between superior endplate of the most tilted vertebra cranially and inferior endplate of the most tilted vertebra caudally. Sagittal measurements included:(1) thoracic kyphosis (TK, T5-T12); (2) pelvic tilt (PT); (3) pelvic incidence minus lumbar lordosis (PI-LL); (4) sagittal vertical axis (SVA); and (5) pelvic incidence (PI).

Because spinal osteotomies were conducted in all patients, osteotomy grades and osteotomy levels were recorded, too. Instrumented levels, distribution of uppermost or lowest instrumented vertebra (UIV or LIV), and levels of interbody fusion were recorded as well.

#### Preoperative coronal pattern evaluation

According to the spatial relationship between C7 PL and major coronal curve on full-spine standing posteroanterior radiographs, the preoperative patterns were classified into two patterns: Pattern 1(concave pattern), C7 PL shifted to the concave side of the major curve; Pattern 2(convex pattern), C7 PL shifted to the convex side of major curve [7]. Three experienced spine surgeons reviewed all radiographs and classified them into two patterns.

According to Obeid et al [8] proposed classification, based on absolute values of GCM preoperatively, patients were classified into 3 types:

Type 0: GCM less than 20 mm plus either Pattern 1 or Pattern 2. To facilitate factorial analysis, patients with type 0 were further subdivided into type 0–1 (GCM less than 20 mm plus Pattern 1) and type 0–2 (GCM less than 20 mm plus Pattern 2).

Type 1: GCM greater than 20 mm plus Pattern 1.

Type 2: GCM greater than 20 mm plus Pattern 2.

Therefore, there were 4 groups (type 0–1, type 0–2, type 1, type 2) involved in this study.

Postoperative coronal imbalance was defined as GCM greater than or equal to 20 mm. Postoperative imbalance/balance ratio was also recorded, which could reflect the incidence of postoperative coronal imbalance.

#### Statistics

Intra-pattern comparison (type 0–1/type 1 or type 0–2/type 2) of continuous variables were conducted using independent t test, continuous variables among 4 groups were compared using one-way analysis of variance (ANOVA). Categorical variables were compared using Chi-square analysis or Fisher’s exact test. To determine the main effects of preoperative pattern and preoperative GCM on the postoperative coronal imbalance, a two-factor ANOVA was used for further factorial analysis. During factorial analysis for postoperative imbalance/balance ratio, postoperative coronal balance was coded as “0”, and postoperative coronal imbalance was coded as “1”. The statistical analysis was performed using SPSS computer software (version 24; SPSS, Chicago, IL, USA). *P* < 0.05 was set as statistical significance.

## Results

### Comparison of patient characteristics and surgical parameters among 4 groups

There was no significant difference regarding sex, age at surgery, instrumented levels, distribution of UIV or LIV, interbody fusion levels, osteotomy grades and levels among the four groups (Table 1).

**Table 1:** Patients’ demographics and surgical parameters in 4 groups

	Pattern 1			Pattern 2			*P* value
groups	type 0–1	type 1	(*P* value)	type 0–2	type 2	(*P* value)	
Patients No	34	21	-	30	27	-	-
Sex(m:f)	4:30	3:18	(0.785)	6:24	3:24	(0.476)	0.753
Age at surgery	63.3 ± 6.0	63.9 ± 6.0	(0.750)	64.4 ± 7.0	62.1 ± 6.9	(0.212)	0.578
Instrumented level	8.9 ± 2.0	9.2 ± 3.5	(0.695)	8.0 ± 2.4	8.6 ± 2.1	(0.390)	0.327
UIV (T10 or above: below	24:10	13:8	(0.505)	15:15	16:11	(0.483)	0.412
LIV (non-pelvic: S1 or below)	11:23	6:15	(0.768)	7:23	4:23	(0.416)	0.447
Interbody fusions	1.6 ± 1.1	1.5 ± 1.2	(0.775)	1.8 ± 1.2	1.7 ± 1.5	(0.943)	0.909
Osteotomy grade	1.9 ± 0.4	1.8 ± 0.4	(0.679)	1.8 ± 0.4	1.8 ± 0.4	(0.858)	0.870
Osteotomy level	3.5 ± 0.9	3.5 ± 0.7	(0.915)	3.2 ± 0.6	3.5 ± 0.8	(0.073)	0.190

*UIV* upper instrumented vertebra, *LIV* lower instrumented vertebra

### Comparison of coronal parameters before and after surgery among 4 groups

As shown in Table 2, there was significant difference regarding preoperative GCM and change in GCM in both intra-pattern 1(concave pattern) and intra-pattern 2(convex pattern) groups, and among 4 groups, too. Significant difference regarding postoperative GCM was seen among 4 groups (*F* = 6.219, *p* = 0.001), although there was no significant difference in either intra-pattern 1(concave pattern) groups (*t* = 0.984, *p* = 0.330) or intra-pattern 2 (convex pattern) groups (t = -0.706, *p* = 0.483); Similarly, significant difference regarding postoperative coronal imbalance/balance ratio existed among 4 groups (*x*^2^ = 22.506, *p* < 0.001), despite no significant difference in either intra-pattern 1(concave pattern) groups (Fisher’s exact test, *p* = 0.696) or intra-pattern 2 (convex pattern) groups (*x*^2^ = 0.970, *p* = 0.325). But significant differences regarding postoperative imbalance/balance ratio existed in preoperatively balanced or imbalanced patients with different patterns (type 0–1 vs. type 0–2, or type 1 vs. type 2) (*x*^2^ = 7.567, *p* = 0.006; *x*^2^ = 14.106, *p* < 0.001, respectively). There was no significant difference in pre- and post-operative major Cobb angle and change in major Cobb angle among 4 groups (Table 2). In addition, no significant difference was seen regarding sagittal parameters before and after surgery among 4 groups (Table 3).

**Table 2:** Coronal parameters before and after surgery in 4 groups

	Pattern 1			Pattern 2			*P* value
groups	type 0–1	type 1	(*P* value)	type 0–2	type 2	(*P* value)	
Patients No.	34	21	-	30	27	-	-
Preoperative major Cobb angle	26.1 ± 15.9	31.4 ± 16.3	(0.241)	24.5 ± 11.9	25.9 ± 13.4	(0.685)	0.391
Postoperative major Cobb angle	8.1 ± 7.3	9.6 ± 7.2	(0.466)	7.9 ± 5.8	9.0 ± 6.3	(0.509)	0.792
Δ major Cobb angle	18.0 ± 12.0	21.8 ± 11.7	(0.254)	16.6 ± 9.5	17.3 ± 10.0	(0.793)	0.371
Preoperative GCM	9.1 ± 5.1	28.8 ± 30.9	(**0.001**)	9.0 ± 5.0	37.6 ± 11.7	(**0.000**)	**0.000**
Postoperative GCM	12.8 ± 11.6	9.6 ± 11.5	(0.330)	20.2 ± 14.1	22.7 ± 12.3	(0.483)	**0.001**
Δ GCM	6.5 ± 17.5	36.2 ± 19.4	(**0.000**)	-11.3 ± 13.9	14.6 ± 17.8	(**0.000**)	**0.000**
Postoperative imbalance:balance	6:28	2:19	(0.696)	15:15	17:10	(0.325)	**0.000**

Boldface indicates statistical significance

*GCM* global coronal malalignment

**Table 3:** Sagittal parameters before and after surgery in 4 groups

	Pattern 1			Pattern 2			*P* value
groups	type 0–1	type 1	(*P* value)	type 0–2	type 2	(*P* value)	
Patients No.	34	21	-	30	27	-	-
Preoperative thoracic kyphosis	14.4 ± 10.4	17.6 ± 11.8	(0.296)	15.3 ± 10.0	12.6 ± 12.3	(0.367)	0.475
Postoperative thoracic kyphosis	22.1 ± 8.3	26.6 ± 9.9	(0.076)	23.7 ± 7.5	20.6 ± 10.1	(0.199)	0.125
Preoperative pelvic tilt	24.8 ± 9.1	23.7 ± 11.1	(0.680)	21.1 ± 11.2	23.9 ± 10.0	(0.334)	0.442
Postoperative pelvic tilt	16.5 ± 7.4	14.8 ± 7.4	(0.389)	15.2 ± 9.2	16.4 ± 8.2	(0.591)	0.825
Preoperative pelvic incidence	44.3 ± 11.0	45.2 ± 9.0	(0.750)	43.5 ± 12.5	45.8 ± 12.2	(0.491)	0.884
Postoperative pelvic incidence	44.4 ± 11.1	45.4 ± 9.2	(0.731)	43.8 ± 12.1	45.3 ± 14.0	(0.665)	0.952
Preoperative PI-LL	22.4 ± 15.3	19.4 ± 14.3	(0.474)	19.4 ± 16.6	21.9 ± 17.6	(0.583)	0.840
Postoperative PI-LL	5.5 ± 9.7	4.2 ± 8.7	(0.593)	5.5 ± 11.9	8.6 ± 11.4	(0.336)	0.509
Preoperative SVA	63.2 ± 49.5	65.9 ± 40.8	(0.835)	63.7 ± 47.5	58.1 ± 37.8	(0.627)	0.937
Postoperative SVA	28.6 ± 18.4	37.3 ± 27.2	(0.162)	31.6 ± 18.6	28.9 ± 19.5	(0.600)	0.441

*PI-LL* pelvic incidence – lumbar lordosis, *SVA* sagittal vertical axis

### Factorial analysis

Since significant differences existed among 4 groups regarding postoperative GCM and imbalance/balance ratio despite no significant intra-pattern differences, two-factor ANOVA was further performed to determine the main effects of preoperative pattern and preoperative GCM on postoperative GCM and imbalance/balance ratio. It revealed that preoperative pattern did exhibit significant effect on postoperative GCM(F_(1,108)_ = 14.286, *p* < 0.001); However, there was no significant effect of preoperative GCM on postoperative GCM (F_(1,108)_ = 0.076, *p* = 0.783); The interaction between preoperative pattern and GCM had no significant effect on postoperative GCM (F_(1,108)_ = 0.687, *p* = 0.409), either. Similarly, preoperative pattern had significant impact on postoperative imbalance/balance ratio (F_(1,108)_ = 30.514, *p* < 0.001), but either GCM or interaction between preoperative pattern and GCM had no effect on postoperative imbalance/balance ratio (F_(1,108)_ = 0.000, *p* = 0.996; F_(1,108)_ = 2.486, *p* = 0.118, respectively) (Table 4).

**Table 4:** Main and interaction effects of preoperative pattern and GCM on postoperative coronal imbalance

	Postoperative GCM				Postoperative imbalance/balance ratio			
	F value	*P* value	Partial η^2^	Observed power	*F* value	*P* value	Partial η^2^	Observed power
Preoperative Pattern	14.286	**0.000**	0.117	0.963	30.514	**0.000**	0.220	1.000
Preoperative GCM	0.076	0.783	0.001	0.059	0.000	0.996	0.000	0.050
Preoperative pattern*GCM	0.687	0.409	0.006	0.130	2.486	0.118	0.023	0.346

Boldface indicates statistical significance

*GCM* global coronal malalignment

## Discussion

The current study showed that preoperative pattern based on the spatial relationship between C7 PL and major curve had significant impact on postoperative GCM or imbalance/balance ratio in DLS patients. However, preoperative GCM alone did not have significant impact on postoperative coronal imbalance, nor did the interaction of preoperative GCM with pattern.

Adult scoliosis encompasses a wide variety of anatomic pathologies and clinical findings, classifying adult scoliosis may be challenging. The classification proposed by Aebi [3] was based only on the etiology of the deformity. SRS classification was based upon radiographic characteristics of deformity, but it did not involve clinical parameters, making it less valuable regarding guidance of treatment [4]. The Schwab-SRS classification system was based mainly upon the impact of sagittal radiographic variables on health status [5]. Recently, based on the absolute value of preoperative GCM (30 mm of GCM set as the threshold), Bao et al. proposed a novel classification and demonstrated that type C deformities were more frequently associated with postoperative persistent coronal imbalance [7]. Obeid et al [8] followed the same philosophy as Bao’s classification and proposed a similar but comprehensive classification using 20 mm of preoperative GCM as threshold of imbalance and adding various modifiers such flexibility of major curve and/or lumbosacral fractional curve. But Obeid’s classification is not experimental design, it is unclear whether Obeid’s classification is practical. Furthermore, the main effects of preoperative GCM and preoperative pattern on the postoperative coronal imbalance, and their interaction effect on the postoperative coronal imbalance are still undetermined.

The current study explored the main effects of preoperative GCM and preoperative pattern on the postoperative coronal imbalance and found that neither preoperative GCM alone nor the interaction of preoperative GCM with pattern had impact on postoperative GCM or imbalance/balance ratio. There was no significant difference regarding postoperative imbalance/balance ratio in either intra-pattern 1 groups or intra-pattern 2 groups, no matter whether these patients were preoperatively coronally balanced or imbalanced. In other words, preoperative GCM, whether balanced or imbalanced, didn’t even have minor effects on postoperative coronal imbalance when 20 mm was set as the threshold of coronal imbalance. This might also imply that from the perspective of avoidance of postoperative coronal imbalance, 20 mm of GCM used as the threshold of coronal imbalance in the preoperative coronal deformity classification might be inappropriate. How many millimeters set as the threshold of coronal imbalance in adult scoliosis is controversial. 20 mm is commonly used as the threshold of coronal imbalance in adolescent idiopathic scoliosis. In adult scoliosis, 30 mm was defined as the threshold value by Bao et al [7], Lowe et al [4] and Choi et al [10] and 40 mm was preferred by Ploumis et al [11]. Further studies are needed to determine how many millimeters is appropriate for the threshold of coronal imbalance.

On the other hand, the preoperative pattern was shown to have significant effect on postoperative coronal imbalance in DLS patients in this study. In other words, it is the preoperative pattern other than preoperative GCM that had the main effects on postoperative coronal imbalance. This made the classification based on preoperative GCM questionable. Moreover, the current study also showed that type 0 (GCM < 20 mm plus either pattern 1 or pattern 2) had higher postoperative imbalance/balance ratio than type 1 (GCM > 20 mm plus pattern 1) did (21:43 vs 2:19, *x*^2^ = 4.345, *p* = 0.037), which suggested that the greater preoperative GCM might not necessarily lead to higher incidence of postoperative coronal imbalance and undermined the basic philosophy of Obeid’s classification. This phenomenon is not alone. A study conducted by Xu et al [12] about risk factors for postoperative coronal imbalance in thoracolumbar congenital kyphoscoliosis using Bao’s classification showed that postoperative imbalance/balance ratio in Type A (GCM < 30 mm plus either pattern 1 or pattern 2) was 12:80 while that in type B (GCM > 30 mm plus pattern 1) was 0:6. These events showed that the basic principle that these two classifications followed might be problematic.

The preoperative pattern having significant impact on postoperative coronal imbalance was also evidenced by other studies. In preoperatively imbalanced patients, Bao et al [7] demonstrated that DLS patients with type C carried greater risk for postoperative coronal imbalance than those with type B, similar results were obtained by Xu et al [12] in patients with thoracolumbar congenital kyphoscoliosis. Furthermore, the current results showed that patients with type 0–2 carried higher postoperative imbalance/balance ratio than those with type 0–1 (15:15 vs 6:28, *x2* = 7.567, *p* = 0.006), which suggested that preoperative pattern still exhibited significant effects on postoperative coronal imbalance even in preoperatively balanced patients. Xu et al [12] wondered why so many postoperative imbalances happened in preoperatively balanced patients, the reason might be that preoperatively balanced patients (Type 0 in Obeid’s classification or type A in Bao’s classification) encompassed two different preoperative patterns (pattern 1 and pattern 2), it was pattern 2 that might lead to surprisingly high incidence of postoperative coronal imbalance even in preoperatively balanced patients. It didn’t matter whether it was preoperative coronal balance or not, it is the pattern that affected the postoperative coronal imbalance. These evidences further put the classification based on the absolute value of GCM in great doubt.

Limitations of this study must be mentioned. Firstly, due to the features of retrospective study, functional scores such as SRS-22 or Oswestry disability index (ODI) were not involved. Secondly, only immediate postoperative coronal imbalance in DLS patients was analyzed in this study, the results of long-term follow-up need to be further explored in the future. Despite these limitations, the current study still demonstrated that preoperative pattern based on the spatial relationship between C7 PL and major curve had significant impact on postoperative coronal imbalance in DLS patients while neither preoperative GCM alone nor the interaction of preoperative GCM with pattern did. This is the first study that demonstrated preoperative coronal pattern had main effects on postoperative coronal imbalance while preoperative GCM did not.

## Conclusion

In DLS patients, it’s the preoperative pattern other than GCM that had main effects on postoperative coronal imbalance. Classification of coronal deformity based on preoperative GCM is questionable. It might be reasonable that the classification of coronal deformity be based on the preoperative pattern other than on the absolute value of preoperative GCM.

## Data Availability

The datasets generated and/or analyzed during the current study are not publicly available but are available from the corresponding author on reasonable request because a few more relevant studies are being done.

## Abbreviations

GCM: Global coronal malalignment
DLS: Degenerative lumbar scoliosis
TLIF: Transforminal lumbar interbody fusion
C7 PL: C7 plumb line
UIV: Upper instrumented vertebra
LIV: Lower instrumented vertebra
CSVL: Central sacral vertical line
SVA: Sagittal vertical axis
TK: Thoracic kyphosis
PT: Pelvic tilt
PI-LL: Pelvic incidence- lumbar lordosis
ODI: Oswestry disability index
